# MDACT: A New Principle of Adjunctive Cancer Treatment Using Combinations of Multiple Repurposed Drugs, with an Example Regimen

**DOI:** 10.3390/cancers14102563

**Published:** 2022-05-23

**Authors:** Richard E. Kast, Alex Alfieri, Hazem I. Assi, Terry C. Burns, Ashraf M. Elyamany, Maria Gonzalez-Cao, Georg Karpel-Massler, Christine Marosi, Michael E. Salacz, Iacopo Sardi, Pieter Van Vlierberghe, Mohamed S. Zaghloul, Marc-Eric Halatsch

**Affiliations:** 1 IIAIGC Study Center, Burlington, VT 05408, USA; 2 Department of Neurosurgery, Cantonal Hospital of Winterthur, 8400 Winterthur, Switzerland; alex.alfieri@ksw.ch (A.A.); marc-eric.halatsch@ksw.ch (M.-E.H.); 3 Naef K. Basile Cancer Center, American University of Beirut, Beirut 1100, Lebanon; ha157@aub.edu.lb; 4 Department of Neurological Surgery, Mayo Clinic, Rochester, MN 55905, USA; burns.terry@mayo.edu; 5 Oncology Unit, Hemato-Oncology Department, SECI Assiut University Egypt/King Saud Medical City, Riyadh 7790, Saudi Arabia; elyamany23@gmail.com; 6 Translational Cancer Research Unit, Dexeus University Hospital, 08028 Barcelona, Spain; mgonzalezcao@oncorosell.com; 7 Department of Neurosurgery, Ulm University Hospital, 89081 Ulm, Germany; georg.karpel@gmail.com; 8 Clinical Division of Medical Oncology, Medical University of Vienna, Waehringer Guertel 18-20, 1090 Vienna, Austria; christine.marosi@meduniwien.ac.at; 9 Rutgers Cancer Institute of New Jersey, New Brunswick, NJ 08901, USA; msalacz@gmail.com; 10 Department of Pediatric Oncology, Meyer Children’s Hospital, Viale Pieraccini 24, 50139 Florence, Italy; iacopo.sardi@meyer.it; 11 Department of Biomolecular Medicine, Ghent University Hospital, Corneel Heymanslaan 10, 9000 Ghent, Belgium; pieter.vanvlierberghe@ugent.be; 12 Children’s Cancer Hospital & National Cancer Institute, Cairo University, Cairo 11796, Egypt; mszagh@yahoo.com

**Keywords:** cholangiocarcinoma, colon cancer, CUSP9v3, glioblastoma, lung cancer, multidrug regimen, repurposing

## Abstract

**Simple Summary:**

We present eight core attributes of cancer growth that we must address for a more effective treatment than we currently have. To do this we outline why a regimen simultaneously using many different drugs will be needed. At our current state of knowledge, even adding two or three drugs will not counter all the growth attributes of a currently incurable cancer. We show in this paper, the details of how an example six drug regimen, when added alongside of current traditional treatments, might inhibit enough of the eight core growth driving elements to allow those standard treatments to be more effective. We further show how medicines from general medical practice used to treat pain, fungal infections, psychosis, leprosy and other non-cancer related illnesses can be repurposed to block cancer cells’ survival pathways and growth drives.

**Abstract:**

In part one of this two-part paper, we present eight principles that we believe must be considered for more effective treatment of the currently incurable cancers. These are addressed by multidrug adjunctive cancer treatment (MDACT), which uses multiple repurposed non-oncology drugs, not primarily to kill malignant cells, but rather to reduce the malignant cells’ growth drives. Previous multidrug regimens have used MDACT principles, e.g., the CUSP9v3 glioblastoma treatment. MDACT is an amalgam of (1) the principle that to be effective in stopping a chain of events leading to an undesired outcome, one must break more than one link; (2) the principle of Palmer et al. of achieving fractional cancer cell killing via multiple drugs with independent mechanisms of action; (3) the principle of shaping versus decisive operations, both being required for successful cancer treatment; (4) an idea adapted from Chow et al., of using multiple cytotoxic medicines at low doses; (5) the idea behind CUSP9v3, using many non-oncology CNS-penetrant drugs from general medical practice, repurposed to block tumor survival paths; (6) the concept from chess that every move creates weaknesses and strengths; (7) the principle of mass—by adding force to a given effort, the chances of achieving the goal increase; and (8) the principle of blocking parallel signaling pathways. Part two gives an example MDACT regimen, gMDACT, which uses six repurposed drugs—celecoxib, dapsone, disulfiram, itraconazole, pyrimethamine, and telmisartan—to interfere with growth-driving elements common to cholangiocarcinoma, colon adenocarcinoma, glioblastoma, and non-small-cell lung cancer. gMDACT is another example of—not a replacement for—previous multidrug regimens already in clinical use, such as CUSP9v3. MDACT regimens are designed as adjuvants to be used with cytotoxic drugs.

**Preface:** gutta cavat lapidem, non vi, sed sæpe cadendo.
**Part One**

## 1. Introduction

Part one of this paper is an overview of eight attributes seen across many common cancers that we interpret as requiring a multidrug approach unless and until a “silver bullet” is found. These attributes are generally well understood in the oncology research community, but bear repeating in a consolidated fashion here. Then, eight principles of a new pharmacological approach—MDACT (multidrug adjuvant cancer treatment), which aims to address many of these attributes—are presented.

Part two of this paper gives specific details of a generalizable six-repurposed-drug regimen that may be considered (gMDACT). Other repurposed multidrug regimens could potentially be constructed with different drugs, or with drugs aiming at a different set of common growth-driving elements. Part two also outlines evidence on how gMDACT may inhibit growth or growth-driving elements active in four representative aggressive human cancers: cholangiocarcinoma, glioblastoma, colon adenocarcinoma, and non-small-cell lung cancer (NSCLC). gMDACT uses the analgesic celecoxib, the antibiotic dapsone, the alcoholism treatment drug disulfiram, the antifungal itraconazole, the antibiotic pyrimethamine, and the antihypertensive telmisartan.

MDACT borrows in principle from several recent repurposing studies in cancer—most prominently from the CUSP9v3 regimen for recurrent glioblastoma, with which MDACT shares three drugs—celecoxib, disulfiram, and itraconazole [1,2,3,4,5,6,7]. Initial clinical reports on CUSP9v3 showed that nine repurposed drugs plus a traditional cytotoxic drug (temozolomide) can be safely given daily over 4+ years if close monitoring and individual dose adjustments are in place [1].

MDACT approaches—and specifically the previous CUSP9v3 regimen and the gMDACT regimen given here—are part of the drug repurposing movement where currently approved and marketed drugs are used in cancer treatment based on their mechanisms of action (MOAs) rather than their approved clinical use [1]. The repurposing movement simply takes a deeper look at what a given medicine does to cellular physiology, rather than how the resultant clinical picture changes.

MDACT follows the injunction of Nguyen et al. that an “…effective treatment for radioresistant glioblastomas may require a cocktail containing multiple agents targeting multiple cancer-inducing pathways in order to have a chance to make a substantial impact on improving the overall glioblastoma survival.” [8].

Starting in 1946 with combining penicillin with sulfadiazine [9], through later combining amoxicillin with clavulanate in the 1980s, to today, combining antibacterial drugs is a well-established practice.

Zapletalova et al. published a regimen (COMBAT) in 2012 using three repurposed drugs—celecoxib, vitamin D, and fenofibrate—with the traditional anticancer drugs temozolomide, etoposide, and retinoic acid [10]. Peryl et al., in 2012, reported a clinical trial using two repurposed drugs—celecoxib and fenofibric acid—to augment five traditional oncology drugs: bevacizumab, thalidomide, etoposide, cyclophosphamide, and cytarabine [11]. In 2016, Rios et al. published a case study using two repurposed drugs—metformin and niacinamide—with the traditional oncology drugs temozolomide, plerixafor, and lapatinib [12]. In 2017, Furuta et al. studied CLOVA—a cocktail that uses four repurposed drugs (cimetidine, lithium, olanzapine, and valproate) with temozolomide [13]. Finally, in 2011, Tatokoro et al. reported on the clinical use of three repurposed drugs—cimetidine, meloxicam, and candesartan—with interferon-alpha [14]. The 3M+Stupp regimen from 2019 of Maraka et al. clinically tested three repurposed non-oncology drugs—memantine, mefloquine, and metformin—as adjuvants to temozolomide, notably without prior testing of the drugs individually [15]. None of the above studies were preceded by individual drug testing or xenograft testing.

A prime example of the multidrug approach that was also not preceded by xenograft study, but is now a well-established treatment for certain lymphomas, is R-CHOP (see discussion of R-CHOP in Section 3.2).

MDACT expands, conceptually consolidates, and follows in the tradition of all of the studies mentioned above.

## 2. Attributes of Cancer Mandating an MDACT Type of Approach

Although generally well known, it bears repeating here that most deadly cancers share the following eight attributes:

1. Spatial and temporal heterogeneity of growth-driving dependencies;
2. Existence of mutually supporting, bilaterally communicating cell communities;
3. Compensatory tumor responses to treatments;
4. Existence of multiple cross-covering, growth-driving signaling pathways functioning in parallel;
5. Metabolic flexibility reliance shifted to another energy source if one becomes inhibited;
6. Pathological engagement of multiple normally functioning body systems to facilitate growth (e.g., cytokines, trophic factors, innervation, interacting stroma, angiogenesis);
7. A subset of tumor stem cells with the potential to enter dormancy.
8. An inverse relationship often seen between growth and invasion, where inhibiting one enhances the other.

Although some cancers can be eliminated by massive cytotoxic assault, most disseminated cancers will not be cured by this approach. Attempting to address more of the eight attributes above might allow a more effective treatment, absent a “silver bullet”. This implies the necessity of a multidrug regimen. MDACT attempts to address our current “…‘one disease–one target–one drug’ dogma [that] obstruct[s] innovation in the most profound manner.” [16]. Multidrug approaches also address tumor heterogeneity between patients (which can also shift over time), thus resulting in a blind precision medicine by default.

The eight principles behind MDACT are discussed below and summarized in Table 1.

## 3. Eight Overarching Principles Driving the Construction of MDACT-Type Regimens

### 3.1. The Principle of Breaking More than One Link in a Chain

If one wishes to stop or impede any chain of events that leads to an unwanted result, that chain should be broken at more than one link. Modern critical machines are designed with this principle in mind, in that failure is averted by requiring several redundant systems to simultaneously fail before the overall function of the machine fails. Modern jet aircraft, for example, have multiple redundant systems—in the event that any one should fail, a second system will fulfill the functions of the failed system. Vulnerable critical systems or machines have many such backup systems, and often a backup to the backups. Competently designed robust machines have compensatory pathways at hand so as to tolerate multiple subsystem failures without major critical end-function dropout. Metastatic cancers tend to be robustly competent, so interrupting a metabolic or growth signaling chain at a single point is too easily repaired, compensated for, or circumvented.

### 3.2. The Principle of Palmer et al.

A second related principle behind the MDACT approach is that of Palmer et al. [17]. CHOP, developed in the late 1970s (and now R-CHOP—rituximab, cyclophosphamide, doxorubicin, vincristine, and prednisone), is a treatment of certain lymphomas [18]. None of these drugs are specific to certain malignant lymphoma clones. However, 87% of lymphomas typically achieve a complete response, with multiyear remissions being common [19]. Not all of the drugs of CHOP or R-CHOP have been tested individually, and no xenograft study preceded clinical trial of the ensemble. Nor would it make sense to decline the combination of amoxicillin and clavulanate because clavulanate did not show any antibacterial effect. This would miss the point of the combination in the same manner as would objections to individually testing the drugs of gMDACT. Individually testing components of the ensemble undercuts the rationale of using the combination.

By analyzing R-CHOP, Palmer et al. concluded that “the 50 years old hypothesis that a curative cancer therapy can be constructed on the basis of independently effective drugs having non-overlapping mechanisms of resistance, without synergistic interaction…has immediate significance for the design of new drug combinations” [17]. Additionally, making parallels with multidrug regimens required to treat tuberculosis, Palmer et al. showed that both original clonal heterogeneity and cytotoxic-drug-driven clonal evolution require a multidrug approach.

Both MDACT and the specific examples of CUSP9v3 and gMDACT engage Palmer et al.’s principle of independent action as well as coordinated actions of drugs (e.g., in shaping operations, vide infra, synthetic cytotoxicity, circumvention prevention actions, CUSP9v3’s Nile Distributary Problem, etc.).

### 3.3. The Chess Principle of Shaping Versus Decisive Operations

Whenever two opposing forces meet—for example, in chess, soccer (football), boxing, or tennis—opponents make a clear distinction between shaping versus decisive operations [20]. Decisive operations are direct efforts to achieve the mission. In chess, this would be to checkmate the opponent’s king. In our case, this would correspond to the use of cytotoxins, surgery, or irradiation to directly kill or remove all cancer cells.

Shaping operations are interventions that set conditions for the decisive operation to be more effective, but do not themselves directly aim to achieve the primary objective. Shaping operations set the stage and prepare the environment to increase the chance that a decisive operation will succeed. In chess, a shaping operation would be to capture one of an opponent’s pieces. With the defenses thus weakened, a checkmating plan—the decisive operation—is more likely to succeed.

Examples of shaping operations in oncology would include rigorous anti-nausea measures to allow the use of highly emetic cytotoxins, the use of mesna to avoid ifosfamide-induced cystitis, forced diuresis for renal protection, etc. Even anesthesia is an oncological shaping operation that allows a decisive operation—surgical removal of a cancer. MDACT-type regimens tend to be largely shaping operations.

### 3.4. The Principle of Chow et al.

In 2021, Chow et al. published an interesting study on the treatment of hypertension [21]. Chow et al. treated arterial hypertension with four different traditional antihypertensive drugs at ¼ the usual dose of each. They used a Ca++ channel blocker (amlodipine), a beta blocker (bisoprolol), a diuretic (indapamide), and an angiotensin receptor blocker (irbesartan) at doses that, when used individually, would not be expected to lower blood pressure much, but did so effectively when all used together, without the side effects characteristic of any of the drugs [21]. MDACT applies this principle to cancer treatment. Granted, we cannot assume that this same method would work in treating cancer as it did in treating hypertension, but it might.

Potential benefits in terms of increased effectiveness with lower side effect burdens make experimental studies of this method worthwhile, remembering that few of us would have predicted the positive results of Chow et al. Others are also considering this translation of Chow et al. to cytotoxic cancer treatments, citing the need to “disrupt paracrine substitutional [growth] signaling” [22].

### 3.5. The Principle of CUSP9v3

CUSP9v3 uses the deeper attributes of ordinary general medicine drugs already in use, seeking to find the intersections between these attributes and identified cancer-growth-driving elements (see previous works for details [1,2,3,4,5,6,7]).

As things stand in 2022, drug repurposing must be a core feature of any regimen that aims to achieve a comprehensive addressing of the many aspects of malignancy as itemized in Section 2 above. Specifically, for intracranial tumors, the drugs chosen must have evidence that they cross the blood–brain barrier. Selection and tailoring of multiple similar cancer-specific drugs is required.

### 3.6. The Chess Aphorism—All Moves Create Strengths and Weaknesses

The concept borrowed from chess is that every move creates weaknesses and strengths. The redundant systems that make aircraft safer also make them heavier and more expensive. An attack in chess, boxing, or fencing creates an opening for the opponent’s counterattack. As applied to the practice of medicine, this means that all of our interventions to cure or ameliorate disease will have aspects of harm inherent to them. These can be trivial or serious. Much of medical research is devoted to determining the relative proportion of harm to benefit. MDACT aims to be conservative, i.e., far towards the low-risk end of that spectrum.

However, as Frederick the Great said, “He who tries to hold onto everything, holds onto nothing”. Accepting some risk is unavoidable if one is to constructively intervene in a disease process. Reluctance to accept harms wrought by a treatment may intrinsically lead to accepting greater harm wrought by the disease. The risk of harm must be commensurate with the threats of the given disease. Commensurate with that dictum, target doses are at the higher end of a standard dosing range, but reduced on an individual basis to eliminate side effects.

As a consequence of this principle, drug selection must put ensuring a low side effect profile first. This will allow greater numbers of drugs to hit a wider array of targets and over longer time periods than we could otherwise with drugs with more burdensome side effects.

### 3.7. The Principle of Mass

This principle is unstated but obvious and accepted throughout medicine. As in the orthopedics aphorism “if a little force didn’t work, maybe more force will”, the principle of mass simply states that by adding mass and force, to a given effort, the chances of achieving the goal increase. Before combat—in chess, military engagements, fencing, football, boxing, and similar clashes of forces—the calculation of mass ratio tends to predict outcomes. Adding mass to one’s effort in such meetings of opposing forces is a well-known maneuver to enhance one’s chances of victory. Mass in this context should be understood as force, or as that which increases inertia.

Applied here, piling on many medicines increases our mass in combating a cancer’s growth. A corollary to this would be, again, that the medicines being piled on must be designed to have fairly low side effect burdens, as in CUSP9v3 and gMDACT.

### 3.8. The Principle of Blocking Parallel Pathways—The Nile Distributary Problem

A cellular consequence of blocking one growth-signaling pathway is often a rerouting of signaling to a parallel pathway, as we see in city traffic or the River Nile Delta. When they exist, an in-parallel flow circuit will take up flow increases, compensating for a blocked route (see discussions of this in the preceding CUSP9v3 papers [1,3,4,6,7]). CUSP9v3 and gMDACT aim to block both in-series growth drives (as in Section 3.1 above) and in-parallel growth-driving circuits.

**Part Two**

## 4. The Drugs of gMDACT

gMDACT is an example of the MDACT pharmacological principle, created with the intent of being applicable across several cancers. gMDACT does not replace CUSP9v3 for glioblastoma, but is perhaps a potential alternative

A winnowing process answered two questions: What are some commonly upregulated growth-facilitating systems across common cancers? What FDA- or EMA-approved drugs already exist that have evidence of being able to inhibit or block them? The result is gMDACT, which is designed to be used with a low-dose cytotoxic drug, e.g., temozolomide.

**Figure 1:**
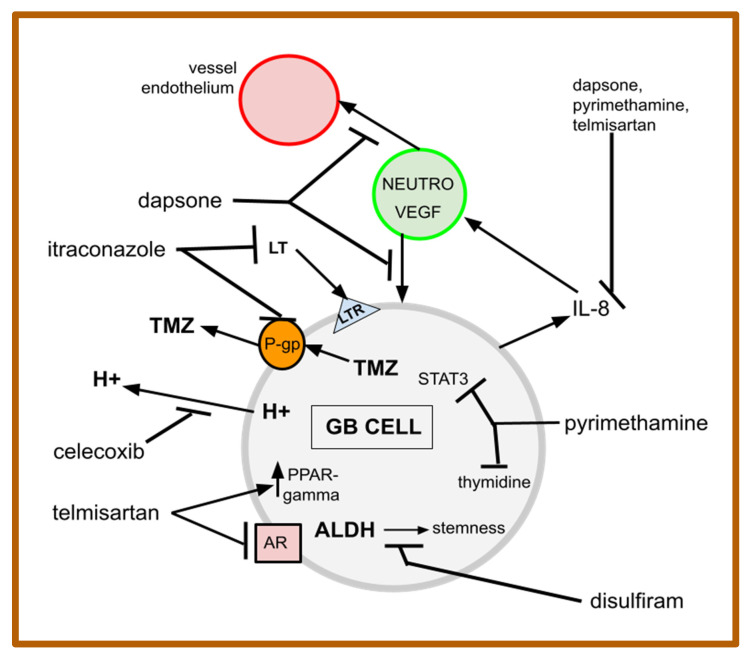
Schematic of some MOAs of gMDACT drugs: The cell here is labeled a glioblastoma (GB) cell, but the schema would be applicable to a variety of cancer cells, including those under discussion here. See the text for details on the growth factors and drugs depicted above. Several MOAs have been omitted for schema clarity; for example, not shown are COX-2 inhibition by celecoxib, Hh inhibition by itraconazole, etc. Pyrimethamine inhibits DHFR catalysis of the reaction dihydrofolate + NADPH + H^+^ ⇆ tetrahydrofolate + NADP^+^, required for methionine and purine synthesis. AR = angiotensin receptor; LT = leukotrienes; LTR = leukotriene receptors; TMZ = temozolomide. The schematic shows drug actions at various levels of abstraction. See the text and references for further details.

The six repurposed drugs of gMDACT are (1) the analgesic drug celecoxib, (2) the antibiotic dapsone, (3) the alcoholism treatment drug disulfiram, (4) the antifungal drug itraconazole, (5) the anti-protozoan drug pyrimethamine, and (6) the antihypertensive telmisartan, together with a continuous low dose of a disease-appropriate classical cytotoxic drug. See Table 2 for an overview of the gMDACT drugs and their putative MOAs. Some of these are depicted in the schematic overview shown in Figure 1.

In general, with multidrug regimens such as gMDACT or CUSP9v3, we aim to give the standard doses as used in the drugs’ non-oncology indication. As with the nine-repurposed-drug regimen CUSP9v3, it should be expected that all people receiving an MDACT-type regimen will require dose reductions based on individual tolerability of one or more of the drugs [1].

### 4.1. Celecoxib

Celecoxib is an older analgesic drug that inhibits two enzymes important in facilitating cancer growth—cyclooxygenase-2 (COX-2), and carbonic anhydrase (CA-IX) [23]. It also inhibits the function of a major drug efflux transporter—P-glycoprotein (P-gp). The P-gp 170-kDa efflux pump (synonymous with ABCB1) is active in cell export of xenobiotics, including chemotherapy drugs such as temozolomide and doxorubicin [24,25,26]. This drug, exporting via upregulated P-gp, becomes a contributor to cancer cells’ resistance to cytotoxic drugs [27,28,29]. Celecoxib reduces cells’ expression of P-gp [30,31,32].

Dozens of recent papers review the hundreds of research studies on the growth-promoting role of prostaglandins in cancer growth generally, and specifically the therapeutic benefits of its synthesis inhibition by COX-2 inhibitors such as celecoxib [33,34,35,36]. The limited clinical benefits of adding celecoxib to standard cancer treatments in previous studies should not discourage its use [36]. Doses used in previous studies were generally far too low (see Reckamp et al. [37]) and, as discussed in part one of this paper, we would not expect any single intervention to be markedly effective, due to the multiple circumvention paths a cancer cell might take.

Reckamp et al. showed data pivotal to the use of celecoxib in treating any cancer. Prostaglandin E2 urinary metabolites in humans only became effectively suppressed at 600 mg of celecoxib twice daily. The nonlinearity of prostaglandin synthesis inhibition by celecoxib should be noted. In humans, after 8 weeks of use, one sees a urinary metabolite decline of only <10% at doses of celecoxib of 200 or 300 mg twice daily, of only 65% after 400 mg twice daily, and 87% at 600 mg twice daily [37]. Therefore, 600 mg of celecoxib twice daily would be the minimum dose in adjunctive treatment of any cancer.

The safety profile of celecoxib differs little from placebo, although large safety studies have investigated lower doses than those of CUSP9v3 or gMDACT [38].

**Figure 2:**
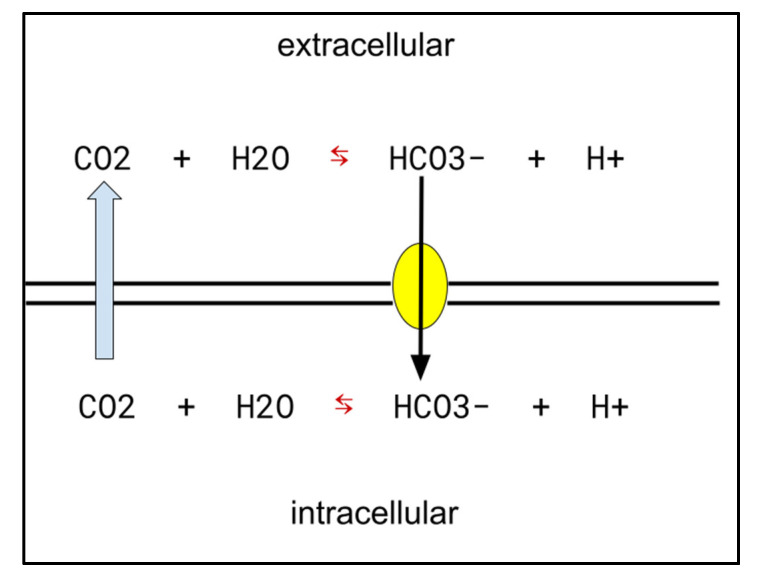
Celecoxib’s CA-IX action in cancer: by inhibiting the interconversion of bicarbonate and CO_2_, depicted by the red arrows, celecoxib reduces cancer cells’ ability to maintain extracellular milieu acidification and maintenance of their intracellular alkaline milieu.

The increased metabolism frequently found in cancer cells requires cell export of the resulting excess H+. Many mechanisms are used in cancers to achieve this export, of which upregulated CA-IX is a major one [39]. CA-IX is an element in maintaining the lower extracellular pH and higher intracellular pH compatible with cancer cells’ survival. This is depicted schematically and simplified in Figure 2. Elevated CA-IX is characteristically found in many cancers where a greater degree of elevation is associated with shorter survival [39,40].

A. Celecoxib in cholangiocarcinoma: High COX-2 expression is associated with shorter cholangiocarcinoma survival [41]. Preclinical studies have shown inhibition of cholangiocarcinoma growth by celecoxib [42,43,44]. Around 92% of cholangiocarcinoma tumors have strong CA-IX immunohistochemistry staining [45].
B. Colon adenocarcinoma: COX-2-driven overproduction of prostaglandin E is an element of dysregulated excess growth across cancers, including colon adenocarcinoma [46,47,48,49]. CA-IX: CA-IX inhibitors increase colon adenocarcinoma cells’ sensitivity to temozolomide and other genotoxic chemotherapies [50]. CA-IX generally tends to be upregulated in hypoxic areas of cancers, and is found specifically in colon adenocarcinoma [51,52,53]. H+ export function by CA-IX has been shown to be crucial for keeping intracellular pH high enough to be compatible with growth in colon adenocarcinoma [54].
C. Celecoxib in glioblastoma: COX-2 and CA-IX are elevated in glioblastoma, and are growth-facilitating elements. The potential usefulness of celecoxib in glioblastoma by inhibiting the function of both of these enzymes was recently reviewed in detail [36]. CA-IX upregulation characteristic of glioblastoma is crucial for this cancer’s adaptation to the hypoxic conditions in which it grows [55].
D. Celecoxib in NSCLC: The roles of elevated COX-2 and CA-IX in NSCLC growth promotion, along with the potential benefit of celecoxib in NSCLC treatment, were recently reviewed [56,57,58]. Several studies show celecoxib’s potential for increasing immune response to NSCLC [59,60]. A recent human clinical immunization study in NSCLC showed that celecoxib enhanced immune responses to a lung cancer lysate vaccine [61]. COX-2 mediates aspects of NSCLC resistance to common traditional cytotoxic drugs, and celecoxib reduces that resistance in experimental models [62,63,64,65,66].

### 4.2. Dapsone

Neutrophils’ tumor trophic function is seen across the common cancers [67,68,69,70,71,72,73,74]. Dapsone is an old sulfone antibiotic that has seen a recent revival of use in treating cancer and nonmalignant neutrophilic dermatoses [75,76,77,78,79]. In treating both the non-malignant dermatoses (e.g., bullous pemphigoid, dermatitis herpetiformis, etc.) and in its anticancer role, dapsone blinds and reduces neutrophils’ homing along an IL-8 gradient. IL-8, synonymous with CXCL8, signals through the receptors CXCR1 or CXCR2, is a neutrophil chemokine, is elevated in the common cancers, and contributes to their associated angiogenesis, and epithelial-to-mesenchymal transition [80,81,82]. In dermatological use, it is established that dapsone inhibits IL-8 function and reduces neutrophil chemotaxis [83,84,85,86]. Dapsone also lowers IL-8 levels in a variety of settings [75,78,87,88,89,90].

Neutrophil-to-lymphocyte ratio (NLR) is elevated across the common cancers, and higher NLR predicts shorter overall survival. Dapsone potentially diminishes some of tumors’ trophic consequences of high NLR by inhibiting neutrophil IL-8 chemotaxis.

Multiple signaling systems converge to upregulate IL-8 [89]. Increased IL-8 goes on to initiate and maintain a growth-promoting role in many cancers, including the four under discussion here.

A. Dapsone in cholangiocarcinoma: IL-8-driven chemotaxis of neutrophils infiltrating cholangiocarcinoma constitutes a trophic function in the growing tumor [91,92,93,94,95,96,97,98,99,100,101]. IL-8 drives angiogenesis, and is elevated in cholangiocarcinoma, where higher levels are associated with shorter survival [102,103,104,105,106,107,108,109,110]. As with other cancers, a higher NLR is strongly associated with shorter survival in cholangiocarcinoma [111].
B. Dapsone in colon adenocarcinoma: Specifically in colon adenocarcinoma, a higher NLR is associated with shorter survival, while a low NLR is associated with longer survival [112,113,114]. Bevacizumab, a pharmaceutical monoclonal antibody to vascular endothelial growth factor (VEGF), is often used in the treatment of colon adenocarcinoma and NSCLC. The benefit of bevacizumab diminishes as the circulating absolute neutrophil count or NLR increases [75,115,116,117,118]. This inverse relationship is due to neutrophils’ delivery of intracellular VEGF, protected from circulating bevacizumab [119,120,121]. IL-8 is actively synthesized by both the malignant cells and their supporting nonmalignant stromata to support growth and angiogenesis in colon adenocarcinoma [122,123,124,125,126,127].
C. Dapsone in glioblastoma: Dapsone’s suppression of IL-8-directed neutrophil chemotaxis and its consequent contributions to glioblastoma growth and angiogenesis were recently reviewed in detail [75]. IL-8 signaling at CXCR2 is a prominent member of the flood of cytokines driving glioblastoma growth [128]. Circulating neutrophils chemotactic to glioblastoma due to IL-8 are an element driving glioblastoma growth and related angiogenesis [129].
D. Dapsone in NSCLC: IL-8 levels are elevated in NSCLC, and a degree of pretreatment elevation is associated with shorter OS [130]. IL-8 elevation in NSCLC is also associated with—and partially drives—increased myeloid-derived suppressor cells [131]. NSCLC tissue, sera, and pleural effusions have increased levels of IL-8 and its receptors, where the degree of elevation is correlated with shorter survival [132,133,134,135,136,137]. As seen commonly in other cancers, an NLR > 4 strongly predicts shorter OS in NSCLC [138,139,140,141,142]. Neutrophil extracellular traps, the presence of which shortens survival in NSCLC, are driven in part by excess IL-8 in NSCLC [68,71].

### 4.3. Disulfiram

Disulfiram is an old alcoholism treatment drug currently used in a wide range of oncology research programs and clinical trials, repurposed for treating cancers. It inhibits aldehyde dehydrogenase (ALDH), resulting in unpleasant accumulation of acetaldehyde if ethanol is consumed. ALDH is a central marker for stemness in both cancer cells and normal cells [143,144,145,146,147,148,149,150]. ALDH might be more properly termed a mediator of stem attributes. Disulfiram deletes or limits ALDH’s mediation of stemness [5,6,7,150,151,152,153,154,155,156]. Empirical in vitro inhibition of multiple cancers’ growth by disulfiram has been demonstrated [157,158]. Disulfiram is currently being investigated in over a dozen human clinical studies as an adjunct in treating cancer stem cell function (clinicaltrials.gov, accessed on 28 February 2022).

A. Disulfiram in cholangioma: The relationship of ALDH with stemness also holds in cholangiocarcinoma, and contributes to its cytotoxic drug resistance [159,160,161,162,163].
B. Disulfiram and colon adenocarcinoma: ALDH is also a core marker/mediator of stemness in colon adenocarcinoma [164,165,166,167,168]. The preclinical activity of disulfiram in inhibition of colon adenocarcinoma cells has been known for over a decade [169,170].
C. Disulfiram in glioblastoma: With potential utility in treating glioblastoma with temozolomide, disulfiram irreversibly inactivates P-gp [171,172,173]. As with other cancers, the ALDH-positive glioblastoma subpopulation has other stem attributes, and is more chemotherapy resistant than the ALDH-negative subpopulation [158,174,175,176].
D. Disulfiram in NSCLC: As it does in other cancers, ALDH contributes to driving stemness and, hence, cytotoxicity resistance, in NSCLC [177,178,179,180,181]. ALDH-driven stemness and chemotherapy resistance in NSCLC are reduced after disulfiram or other inhibitors of ALDH [179,181,182,183].

### 4.4. Itraconazole

Itraconazole is a generic broad-spectrum antifungal drug that is also seeing a renaissance in anticancer applications [184,185,186,187,188,189]. It has three attributes that recommend its use during cancer treatment: (1) it inhibits Hedgehog (Hh) signaling [189,190,191,192,193], (2) it inhibits 5-lipoxygenase (5-LO) [193,194,195,196], and (3) it inhibits the P-gp efflux pump [188,197,198,199].

Hh signaling is essential for embryological development, but is often inappropriately engaged to drive malignant growth [200,201,202,203]. Hh is a common marker/mediator of stemness across cancers [148,204,205]. Hh signaling has a potentially felicitous relationship with pyrimethamine (vide infra) in that low folate states activate compensatory Hh signaling in colon adenocarcinoma [206].

A branch point in arachidonic acid metabolism leads either to the prostaglandin pathway via COX-1/COX-2 or the leukotriene pathway via 5-LO. Itraconazole’s inhibition of 5-LO makes it a particularly good partner drug for COX-2 inhibitors such as celecoxib.

A. Itraconazole in cholangiocarcinoma: 5-LO and its leukotriene products contribute to the growth of cholangiocarcinoma [207,208,209]. Hh signaling is a core growth-driving element in cholangiocarcinoma, and prominently so in the stem subset [210,211,212,213,214,215]. 5-LO is one of the drivers of both myeloid-derived suppressor cell immunosuppression and stemness in cholangiocarcinoma [216].
B. Itraconazole in colon adenocarcinoma: Colon adenocarcinomas overexpress 5-LO, as well as COX-2 [217,218,219]. As found in other cancers, breast cancer’s overexpression of both COX-2 and 5-LO is associated with enhanced aggressiveness [220]. Dual inhibition of COX-2/5-LO inhibits colon cancer proliferation, migration, and invasion to a greater degree than either alone. Specifically, inhibition of 5-LO increases celecoxib’s cytotoxicity to colon cells [221,222,223]. Coordinated participation of COX-2 and 5-LO in carcinogenesis and cancer growth is recognized in several common cancers [220,221,224,225,226,227]. Hh signaling is a major growth-driving element in a variety of cancers, including colon adenocarcinoma [228]. Itraconazole interferes with colon cancer’s cytotoxicity resistance and growth by inhibiting Hh [229,230,231]. Hh signaling is a major driver of colon adenocarcinoma growth [232]. Itraconazole’s inhibition of Hh inhibits colon adenocarcinoma growth [229].
C. Itraconazole in glioblastoma: The rationale for the use of itraconazole during glioblastoma treatment is based on its attributes of Hh inhibition, leukotriene signaling reduction, and reduction in P-gp-mediated cell export of temozolomide, as outlined in the three preceding CUSP9 papers [1,2,3,6]. 5-LO-generated leukotrienes promote glioblastoma migration, growth, and stem attributes [233].
D. Itraconazole in NSCLC: A 2013 study showed that itraconazole plus pemetrexed in NSCLC doubled progression-free survival (PFS) and gave a fourfold increase in overall survival (OS) [234]. In 2017, three reviews of itraconazole’s attributes were published, suggesting its usefulness in interfering with cancer cells’ growth—two in general, and one specifically in NSCLC [235,236,237]. In 2018, Lee et al. outlined the potential of inhaled itraconazole to inhibit NSCLC growth [238]. In 2019, itraconazole was reformulated for superior pharmacokinetics in NSCLC treatment [239]. NSCLC patients given 300 mg of itraconazole orally, twice daily, for two weeks prior to surgery, had decreased tumor volume and reduced vascularity [240]. 5-LO-generated leukotrienes promote NSCLC migration and growth [233,241].

### 4.5. Pyrimethamine

Pyrimethamine is a 248 Da lipophilic drug used to treat malaria for over 50 years, and continues in this role to this day. Three pharmacodynamic attributes of pyrimethamine recommend its use in disseminated human cancer: (1) inhibition of human dihydrofolate reductase (DHFR), (2) inhibition of thymidine phosphorylase, and (3) inhibition of STAT3. Pyrimethamine is seeing increasing adjunctive use in treating cancer [242,243,244].

Pyrimethamine’s Ki = 38 nM at DHFR is comparable to that of the archetypal DHFR inhibitor methotrexate (MTX) (Ki = 2.3 nM) or folinic acid (Ki = 320 nM) and folic acid (Ki = 830 nM) [245,246]. DHFR catalyzes NADPH-dependent reduction of dihydrofolate to tetrahydrofolate. MTX is a high-affinity inhibitor of DHFR commonly used in treating several cancers, which blocks DNA synthesis by disrupting metabolism of methionine, S-adenosyl-methionine, purines, and thymidylate.

The thymidine synthetic pathway depends on the methylation of deoxyuridine, the methyl donor being methylenetetrahydrofolate [247,248,249,250]. The standard DHFR inhibitor used for over 50 years in cancer treatment has been MTX [251,252].

Pyrimethamine is readily exported by P-gp [245]. Thus, the three P-gp-inhibiting drugs of gMDACT—celecoxib, disulfiram, and itraconazole—have the potential to augment pyrimethamine’s effects.

In an acute myelogenous leukemia model, pyrimethamine was more effective in inhibiting growth than MTX. In vitro proliferation was reduced 2.5-fold by pyrimethamine at 0.1 µM, and 12.7-fold at 0.5 µM [253]. Clinically, several patients with polycythemia rubra vera and essential thrombocythemia were successfully controlled with pyrimethamine, as reported in 1987 [254]. It is unclear why early reports in the 1970s of successful pyrimethamine treatment (2 mg/kg/day for 7 days) of meningeal recurrence of acute lymphoblastic leukemia in children have not been followed up, or why such use is currently rare to nonexistent [255].

Pyrimethamine also inhibits mammalian thymidine phosphorylase [256,257]. Thymidine phosphorylase catalyzes the following reaction:

Thymidine + phosphate ⇆ thymine + 2-deoxy-alpha-D-ribose 1-phosphate.

Thymidine phosphorylase activity enhances NF-κB-mediated IL-8 expression in a variety of settings [258,259,260,261].

Pyrimethamine is an inhibitor of STAT3 phosphorylation [262,263,264,265,266,267]. STAT3 is a cytosolic signaling hub, at the middle point of a signaling chain from cell surface receptors to the nucleus. STAT3’s activation by surface growth-stimulating receptors results in STAT3’s phosphorylation, dimerization, and subsequent transport to the nucleus, where it acts as a transcription factor for growth systems identified across cancers [268,269,270]. Many upstream signaling pathways converge on STAT3 to activate it. Pyrimethamine’s STAT3-inhibitory effects are traceable to reduced intracellular folate from DHFR inhibition [266].

The STAT3 signaling hub is overactive in many cancers, including the four under discussion here [271,272,273,274,275]. Of additional potential benefit of STAT3 inhibition, STAT3 activation is an element in myeloid-derived suppressor cells’ function, where inhibiting STAT3 activation was found to reduce myeloid-derived suppressor cell function [275,276].

A. Pyrimethamine in cholangiocarcinoma: In cholangiocarcinoma, upregulated thymidine phosphorylase also contributes to chemotherapy resistance, and furthers survival [109,277,278,279]. Thymidine phosphorylase overexpression enhances growth and suppresses apoptosis in human umbilical vein endothelial cells, as well as increasing VEGF, IL-8, and the growth of cholangiocarcinoma cells [109]. STAT3 activation is an identified growth driver in cholangiocarcinoma [280,281,282].
B. Pyrimethamine in colon adenocarcinoma: As commonly found in other cancers, STAT3 overactivation also constitutes a driving force in colon adenocarcinoma, and particularly so in the stem subpopulation [282,283,284,285,286,287,288]. Multiple experimental, non-marketed inhibitors of STAT3 reduced colon cancer growth in preclinical models [289,290,291]. Growth of colon cancer cells is suppressed when DNA binding of activated STAT3 is prevented [292].
C. Pyrimethamine in glioblastoma: Of great interest for potential use in treating glioblastoma or brain metastases from breast or lung cancer is the unusual property of pyrimethamine in being concentrated in the brain at several times greater levels than in plasma [293,294]. Pyrimethamine is synergistically cytotoxic with temozolomide—the mainstay in current glioblastoma treatment—in melanoma and pituitary adenoma cell lines [295,296]. Glioblastomas have a greatly upregulated thymidine phosphorylase content and activity [297]. Experimental (non-marketed) thymidine phosphorylase inhibitors have no cytotoxicity alone, but are synergistic with temozolomide against glioblastoma cell lines [297].
D. Pyrimethamine in NSCLC: STAT3 is also an active signaling hub identified in NSCLC growth [298]. A preclinical study showed in vitro and xenograft growth inhibition of NSCLC by pyrimethamine [257,258,299].

### 4.6. Telmisartan

Telmisartan is a generic angiotensin-receptor-blocking drug (ARB) used to treat hypertension. Angiotensin-converting enzyme (ACE) converts angiotensin I to the eight-amino-acid peptide angiotensin II. Angiotensin II signals widely throughout the body, mostly to increase blood pressure. Investigation of the potential of ARBs to inhibit cancer’s growth in general has a long history [300]. ACE and ACE-related signaling are recognized drivers across the common cancers [301,302]. A second attribute of telmisartan is its stimulation of PPAR-γ [302,303,304,305].

Patients being treated for hypertension with telmisartan show decreased IL-8 [306].

A. Telmisartan in cholangiocarcinoma: Telmisartan triggers cholangiocarcinoma G0/G1 cell-cycle arrest in vitro [307]. Telmisartan also triggers cell-cycle arrest in a wide variety of gastrointestinal and other common cancers [308,309,310,311,312,313,314,315,316,317,318]. ACE and ACE-related signaling are active specifically as elements driving cholangiocarcinoma growth [319,320,321].
B. Telmisartan in colon adenocarcinoma: Telmisartan blocks angiotensin II receptor type 1. Marketed to treat hypertension, it has several other attributes and uses. Colon adenocarcinoma cells express angiotensin II receptor 1. Telmisartan’s IC_50_ to several colon cancer cell lines in vitro is between 1 and 5 µM [322]. Irbesartan, a marketed pharmaceutical ARB similar to telmisartan, inhibits colitis-associated colon cancer development [323]. Candesartan, another pharmaceutical ARB similar to telmisartan, inhibits colon adenocarcinoma xenograft growth and tumor-related fibrosis [324]. Other studies have found that colon cancer cell growth inhibition is greater with telmisartan compared to candesartan [325]. Candesartan decreased the immune suppression function of tumor-associated CD11b+ T cells and decreased their production of VEGF and arginase, and increased interferon-γ synthesis in the lymph nodes of colon-cancer-bearing mice, without having effect on in vivo tumor growth [326].
C. Telmisartan in glioblastoma: Telmisartan was cytotoxic via peroxisome proliferator-activated receptor gamma (PPAR-γ) agonism in glioblastoma cells in vitro, at low concentrations [327]. Telmisartan-induced inhibition of glioblastoma growth via angiotensin receptor inhibition has been extensively reviewed previously [328]. PPAR-γ is upregulated in mesenchymal glioblastoma stem cells, with agonism suppressing growth [329].
D. Telmisartan in NSCLC: Several studies show longer survival in NSCLC in patients receiving an ARB [330,331,332,333]. This effect, although slight, has been consistently found across studies. Empirically, telmisartan inhibits experimental NSCLC growth [334]. Various putative MOAs for telmisartan’s inhibition of NSCLC have been identified [334,335,336,337,338,339].

## 5. Conclusions

As in point number 3 in Table 1, MDACT-type regimens are designed to be shaping operations more than decisive operations. MDACT regimens are designed primarily to delay the development of resistance and impede tumor regrowth, and to limit normal growth-driving signals that are abnormally engaged by a malignant tumor to further its growth. MDACT regimens aim to set the conditions for cytotoxic medications to be more active, to prepare malignant cells for a cytotoxin by defeating some of the signaling systems responsible for their hardiness.

Part one of this paper outlined what we are up against in trying to treat human cancer—stromata’s trophic functions, engagement of remote physiological systems (e.g., bone marrow, adrenal, thyroid, neural inputs to tumors, etc.), immunological/inflammatory contributions to tumor growth, immunological/inflammatory contributions to tumor elimination, interacting malignant cell communities within the main tumor, the evolution of those communities, entry to a treatment-resistant quiescent state, and other currently unknown elements driving discrepancies between lab results and clinical disease. Thus, there is a lot to consider.

The MDACT approach aims to address as many of these tumor growth and survival elements as possible with already-marketed, non-oncology drugs to augment the effects of traditional cytotoxic, kinase-inhibitory, or other traditional current oncology drugs. Clinical experience with CUSP9v3 and other similar preceding regimens shows that this can be done safely over years of treatment [1,340]. The risks of unanticipated drug–drug interaction are mitigated by gradual one-by-one drug addition at low doses over several weeks, followed by several weeks of gradual dose up-titration. As shown by Chow et al. in hypertension and Halatsch et al. in CUSP9v3 treatment of glioblastoma, multidrug regimens can be given safely if three conditions are met: (1) careful pharmacological evaluation during drug selection to obviate known interaction potential, (2) adding drugs one at a time with weekly appropriate lab and clinical evaluations, and (3) gradual dose up-titration [1,3,6,7,21]. Related to this, we expect in any MDACT-type regimen that individual patients will require dose reduction from target doses of one or more of the drugs, as was the case in the CUSP9v3 trial [1]. Target doses for MDACT-type regimens are the upper end of the standard doses for the individual drugs when used in their non-oncology setting, with certain physiologically based exceptions, e.g., for celecoxib.

Part two of this paper gave an example of a six-repurposed-drug regimen, gMDACT, which might be applicable across a range of cancers. The six gMDACT drugs—celecoxib, dapsone, disulfiram, itraconazole, pyrimethamine, and telmisartan—interfere with multiple growth-driving elements commonly elevated in common cancers. gMDACT does not replace CUSP9v3.

Multiple synergies between the gMDACT drugs have been mentioned and referenced in the text above. (1) Three of the gMDACT medicines (celecoxib, disulfiram, and itraconazole) inhibit P-gp. (2) Augmentation of temozolomide’s cytotoxicity to various cancer cell types has been either empirically demonstrated or predicted by theory for four of the gMDACT drugs (celecoxib, itraconazole, pyrimethamine, and telmisartan), making low-dose metronomic temozolomide a potentially good choice of cytotoxic drug with gMDACT. (3) Four of the gMDACT drugs (celecoxib, dapsone, pyrimethamine, and telmisartan) have shown the potential to downregulate IL-8 synthesis.

We have wide clinical non-oncology experience with the gMDACT drugs. They are low-risk for adverse events. The gMDACT mix of drugs might be applicable adjuvants to a broad range of common cancers.

## Figures and Tables

**Table 1:** Overview of the eight pharmacology approaches, woven together to formulate an MDACT-type regimen.

1. The principle of breaking more than one link in any chain in a series that leads to undesired outcomes.
2. The principle of Palmer et al., of achieving fractional cell killing with multiple drugs with independent MOAs.
3. The principle of shaping versus decisive operations—both required for a successful cancer treatment, with MDACT regimens being largely shaping operations.
4. A principle adapted from Chow et al. of using multiple simultaneous cytotoxic medicines at low doses.
5. As in CUSP9v3, the principle of using non-oncology drugs from general medical practice, repurposed to block multiple survival paths.
6. The concept borrowed from chess that every move (i.e., medical or other intervention) creates weaknesses and strengths.
7. The principle of mass, where inadequate response is redressed by simply adding more force.
8. The Nile Distributary Problem, where the existence of parallel growth-driving pathways allows signaling flow to proceed when a given pathway is blocked.

**Table 2:** Overview of the gMDACT regimen.

Drug	Dose	Usual Use—Target Use in gMDACT
Celecoxib	600 mg × 2	Analgesic—COX-2, CA-IX, P-gp
Dapsone	100 mg × 2	Antibiotic—neutrophils, IL-8, VEGF
Disulfiram	250 mg × 2	Anti-alcoholism—ALDH, P-gp
Itraconazole	200 mg × 2	Antifungal—Hh, 5-LO, P-gp
Pyrimethamine	50 mg × 1	Antibiotic—STAT3, DHFR, IL-8, thymidine phosphorylase
Telmisartan	80 mg × 1	Anti-hypertensive—PPAR-gamma, ARB, IL-8

Target doses are down-titrated to mitigate any unpleasant side effects or lab abnormalities should these occur. References in text. ALDH = aldehyde dehydrogenase; ARB = angiotensin receptor blocker; CA = carbonic anhydrase; COX-2 = cyclooxygenase-2; DHFR = dihydrofolate reductase; Hh = hedgehog; 5-LO = 5-lipoxygenase; P-gp = p-glycoprotein efflux pump, synonymous with ABCB1. With respect to dose suggestions, these are ideal target doses. Many people will require dose reductions from this ideal due to side effects or adverse reactions.

## Data Availability

The authors are happy to provide further references to matters discussed in this work. Contact corresponding author R.E.K.

## Abbreviations

ACE	Angiotensin-converting enzyme
ALDH	Aldehyde dehydrogenase
ARB	Angiotensin receptor-blocking drug
CA	Carbonic anhydrase
COX-2	Cyclooxygenase-2
DHFR	Dihydrofolate reductase
Hh	Hedgehog
5-LO	5-Lipoxygenase
MTX	Methotrexate
MOA	Mechanism of action
MDACT	Multidrug adjuvant for cancer treatment
NLR	Neutrophil-to-lymphocyte ratio
VEGF	Vascular endothelial growth factor
